# Three Novel and One Potential Hotspot *CPT1A* Variants in Chinese Patients With Carnitine Palmitoyltransferase 1A Deficiency

**DOI:** 10.3389/fped.2021.771922

**Published:** 2021-11-12

**Authors:** Weifeng Zhang, Yanru Chen, Chunmei Lin, Weilin Peng, Qingliu Fu, Yiming Lin

**Affiliations:** ^1^Department of Neonatology, Quanzhou Maternity and Children's Hospital, Quanzhou, China; ^2^Center of Neonatal Disease Screening, Quanzhou Maternity and Children's Hospital, Quanzhou, China

**Keywords:** newborn screening, carnitine palmitoyltransferase 1A deficiency, *in silico* prediction, *CPT1A* gene, tandem mass spectrometry

## Abstract

Carnitine palmitoyltransferase 1A (CPT1A) deficiency is an inherited disorder of mitochondrial fatty acid β-oxidation that impairs fasting ketogenesis and gluconeogenesis in the liver. Few studies implementing newborn screening (NBS) for CPT1A deficiency in the Chinese population have been reported. This study aimed to determine the biochemical, clinical, and genetic characteristics of patients with CPT1A deficiency in China. A total of 204,777 newborns were screened using tandem mass spectrometry at Quanzhou Maternity and Children's Hospital between January 2017 and December 2018. Newborns with elevated C0 levels were recruited, and suspected patients were subjected to further genetic analysis. Additionally, all Chinese patients genetically diagnosed with CPT1A deficiency were reviewed and included in the study. Among the 204,777 screened newborns, two patients were diagnosed with CPT1A deficiency; thus, the estimated incidence in the selected population was 1:102,388. In addition to the two patients newly diagnosed with CPT1A deficiency, we included in our cohort 10 Chinese patients who were previously diagnosed. Five of these 12 patients were diagnosed *via* NBS. All patients exhibited elevated C0 and/or C0/(C16+C18) ratios. No clinical symptoms were observed in the five patients diagnosed *via* NBS, while all seven patients presented with clinical symptoms, including fever, cough, vomiting, diarrhea, and seizures. Eighteen distinct *CPT1A* variants were identified, 15 of which have been previously reported. The three novel variants were c.272T>C (p.L91P), c.734G>A (p.R245Q), and c.1336G>A (p.G446S). *in silico* analysis suggested that all three novel variants were potentially pathogenic. The most common variant was c.2201T>C (p.F734S), with an allelic frequency of 16.67% (4/24). Our findings demonstrated that NBS for CPT1A deficiency is beneficial. The three novel variants expand the mutational spectrum of *CPT1A* in the Chinese population, and c.2201T>C (p.F734S) may be a potential hotspot *CPT1A* mutation.

## Introduction

Carnitine palmitoyltransferase 1A (CPT1A) (OMIM 255120; EC 2.3.1.21) deficiency is an inherited autosomal recessive disorder of mitochondrial fatty acid β-oxidation that impairs fasting ketogenesis and gluconeogenesis in the liver. The causative gene, *CPT1A* (OMIM 600528), is located on chromosome 11q13.1-q13.2. Patients may present with a variety of symptoms, including seizures, lethargy, hypoglycemia, vomiting, diarrhea, fever, and hepatomegaly triggered by fasting. Sudden infant death and hypoketotic hypoglycemia have been reported in patients with severe deficiency (1).

Tandem mass spectrometry (MS/MS) has been widely used in newborn screening (NBS) for inherited metabolic diseases. CPT1A deficiency can be detected by MS/MS, with free carnitine (C0) and C0 to free long-chain acylcarnitine [C0/(C16+C18)] as the primary screening markers (2, 3). As early identification and timely treatment can prevent clinical symptoms, NBS is an important tool for diagnosing CPT1A deficiency.

Several patients have been reported worldwide since the first description of this disorder in 1981 (4). In particular, a high prevalence of CPT1A deficiency was observed in the Inuit, First Nations, and Alaska Native populations (5–8). However, few studies have used NBS to diagnose CPT1A deficiency in the Chinese population (9–13). In this study, we used NBS to diagnose CPT1A deficiency in a southern Chinese population. Furthermore, we systematically reviewed Chinese patients previously diagnosed with CPT1A deficiency to improve our understanding of this rare disorder.

## Materials and Methods

### Study Population

A total of 204,777 newborns were screened by MS/MS at Quanzhou Maternity and Children's Hospital between January 2017 and December 2018, and newborns with elevated C0 levels (C0 > 60 μmol/L, cut-off value: 10–60 μmol/L) were recruited for the study. Additionally, all Chinese patients previously genetically diagnosed with CPT1A deficiency were reviewed and included in the study. Data from these patients were retrieved from PubMed (http://www.ncbi.nlm.nih.gov/pubmed) by searching the keywords “carnitine palmitoyltransferase 1A deficiency” or “CPT1A deficiency,” “Chinese” or “China,” and “*CPT1A*.” Written informed consent was obtained from the parents of all patients. This study was approved by the Ethical Committee of Quanzhou Maternity and Children's Hospital. Written informed consent was obtained from the parents of all patients for the collection of samples and the publication of medical data.

### NBS for CPT1A Deficiency

Sample collection, delivery, and pre-processing were performed as described in our previous article (14). Amino acids and acylcarnitines were quantitated using an ACQUITY TQD mass spectrometer (Waters, Milford, MA, USA). Newborns with elevated C0 levels (cut-off value: 10–60 μmol/L) and/or C0/(C16+C18) ratios (cut-off value: 0–40) were recalled. Those who tested positive were subjected to genetic testing.

### Genetic Analysis

Targeted next-generation sequencing (NGS) was performed as previously described (15). A multigene panel covering 94 genes associated with inherited metabolic disorders (including *CPT1A*) was applied. All candidate variants identified by NGS were validated using Sanger sequencing. *CPT1A* exons and flanking intron sequences were amplified by polymerase chain reaction (PCR) under standard conditions. Primer sequences are available upon request. PCR products were sequenced directly using an ABI Prism 3500 Genetic Analyzer (Applied Biosystems, Foster City, CA, USA). The sequencing results were analyzed using Chromas 2.6.5 (Technelysium Pty Ltd, Australia), and the sequences were aligned using the *CPT1A* transcript (NM_001876). DNAMAN 8 (Lynnon Biosoft, San Ramon, CA, USA) was used to identify nucleotide variants.

All candidate variants were searched in several frequently used databases, as previously described (15). The pathogenicity of the novel missense variants was predicted using several *in silico* tools, including SIFT, PolyPhen-2, PROVEAN, and MutationTaster. Evolutionary conservation was analyzed using ClustalX (http://www.clustal.org/clustal2), and homology modeling was performed using the Swiss Model Workspace to analyze changes in the three-dimensional (3D) structure. Finally, all novel variants were evaluated based on the standard recommendations and guidelines for sequence variant interpretation, as published by the American College of Medical Genetics and Genomics and the Association for Molecular Pathology (ACMG/AMP) (16).

## Results

### NBS for CPT1A Deficiency

Of the 204,777 screened newborns, 199 exhibited elevated C0 levels during initial NBS, of which 3 had extremely high C0 levels and 18 were accompanied by elevated C0/(C16+C18) ratios. During the recall stage, 12 newborns had elevated C0 levels, and 6 had elevated C0/(C16+C18) ratios. Two patients were finally diagnosed with CPT1A deficiency; thus, the incidence in the selected population was estimated to be 1:102,388.

### Biochemical and Clinical Characteristics

In addition to the two patients newly diagnosed with CPT1A deficiency, we included in our cohort 10 Chinese patients who were previously diagnosed. Five of these 12 patients were diagnosed *via* NBS. All 12 patients exhibited elevated C0 levels; 11 patients (91.7%) also exhibited elevated C0/(C16+C18) ratios. The median C0 concentration and C0/(C16+C18) ratio were 136.00 ± 39.13 μmol/L (range: 79.65–193.61 μmol/L, cut-off value: 10–60 μmol/L) and 840.55 ± 513.80 (range: 34.49–1,595.54 μmol/L, cut-off value: 0–40), respectively.

No clinical symptoms were observed in the five patients diagnosed *via* NBS. The follow-up period of patients (no. 1–3) were between 2 and 3 years, while follow-up data of patients (no. 9 and 10) were not available. In contrast, all seven patients presented with clinical symptoms, including fever, cough, vomiting, diarrhea, and seizures. The age of onset ranged from 6 months to 1 year and 10 months. Detailed information on the biochemical and clinical manifestations of these patients is presented in Table 1.

**Table 1:** Biochemical, clinical, and molecular features of 11 Chinese patients with CPT1A deficiency.

**Patient**	**Sex**	**Type**	**Acylcarnitine analysis**		**Genotype**		**Onset**	**Clinical manifestions**	**References**
			**C0**	**C0/(C16+C18)**	**Allele 1**	**Allele 2**			
1	F	NBS	113.46	34.49	**c.734G>A (p.R245Q)**	**c.1336G>A (p.G446S)**	None	None	This study
2	M	NBS	79.65	379.29	c.1131G>C (p.E377D)	**c.272T>C (p.L91P)**	None	None	This study
3	M	NBS	128.1	512.4	c.1318G>A (p.A440T)	c.2201T>C (p.F734S)	None	None	(11)
4	F	SMS	179.68	499.5	c.1846G>A (p.V616M)	c.2201T>C (p.F734S)	1 y, 10 m	Seizure	(13)
5	M	SMS	191.5	1,473	c.693+1G>A	c.967+81C>T	1 y, 2 m	Fever, vomiting, diarrhea, seizure	(12)
6	M	SMS	144.9	920	c.968-8C>T	c.946C>T (p.R316W)	1 y, 2 m	Fever, vomiting, diarrhea, seizure	(12)
7	M	SMS	160.9	1,641	c.1787T>C (p.L596P)	c.2201T>C (p.F734S)	8 m	Fever, seizure, hypoglycemia	(12)
8	F	SMS	111.6	558	c.1131G>C (p.E377D)	c.124_126delAAG (p.K42del)	6 m	Fever, seizure, hypoglycemia	(12)
9	M	NBS	107.4	571	c.281+1G>A	c.956C>T (p.G319V)	None	None	(12)
10	M	NBS	84.3	843	c.740C>T (p.P247L)	c.577delC (p.M194*)	None	None	(12)
11	F	SMS	193.61	1,595.54	c.281+1G>A	Deletion of exon 15-18	1 y, 6 m	Fever, diarrhea, seizure	(10)
12	M	SMS	136.88	1,059.37	c.1787T>C (p.L596P)	c.2201T>C (p.F734S)	8 m	Fever, cough, diarrhea, seizure	(9)

*M, male; F, female; NBS, newborn screening; SMS, selective metabolic screening; y, years; m, months; cut-off value, C0: 10–60 μmol/L, C0/(C16+C18): 0–40; the novel CPT1A variants are in boldface type*.

### Genetic Findings

**Figure 1:**
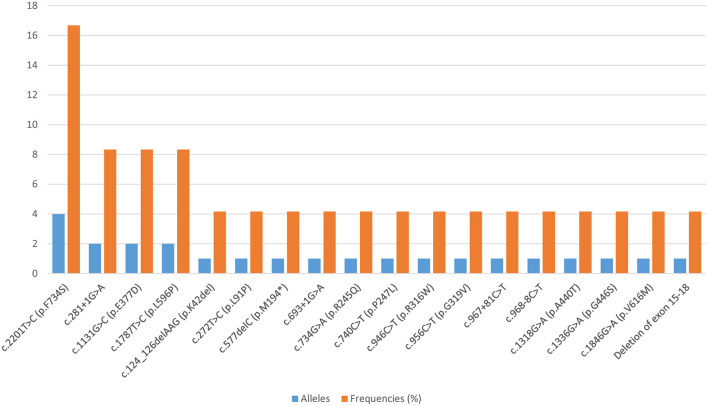
Overview of all *CPT1A* variants found in the Chinese population.

All patients harbored compound heterozygous *CPT1A* variants. Eighteen distinct variants were identified, among which 61.11% (11/18) were missense variants, 22.22% (4/18) affected splicing, 11.11% (2/18) were frameshift variants, and 5.56% (1/18) were large deletions. Fifteen of these *CPT1A* variants were previously reported, and the other three were novel. The novel variants were c.272T>C (p.L91P), c.734G>A (p.R245Q), and c.1336G>A (p.G446S). The most common variant was c.2201T>C (p.F734S), with an allelic frequency of 16.67% (4/24). Relatively common variants were c.281+1G>A, c.1131G>C (p.E377D), and c.1787T>C (p.L596P), which were each identified twice. Each of the remaining 14 variants was identified only once (Figure 1).

**Figure 2:**
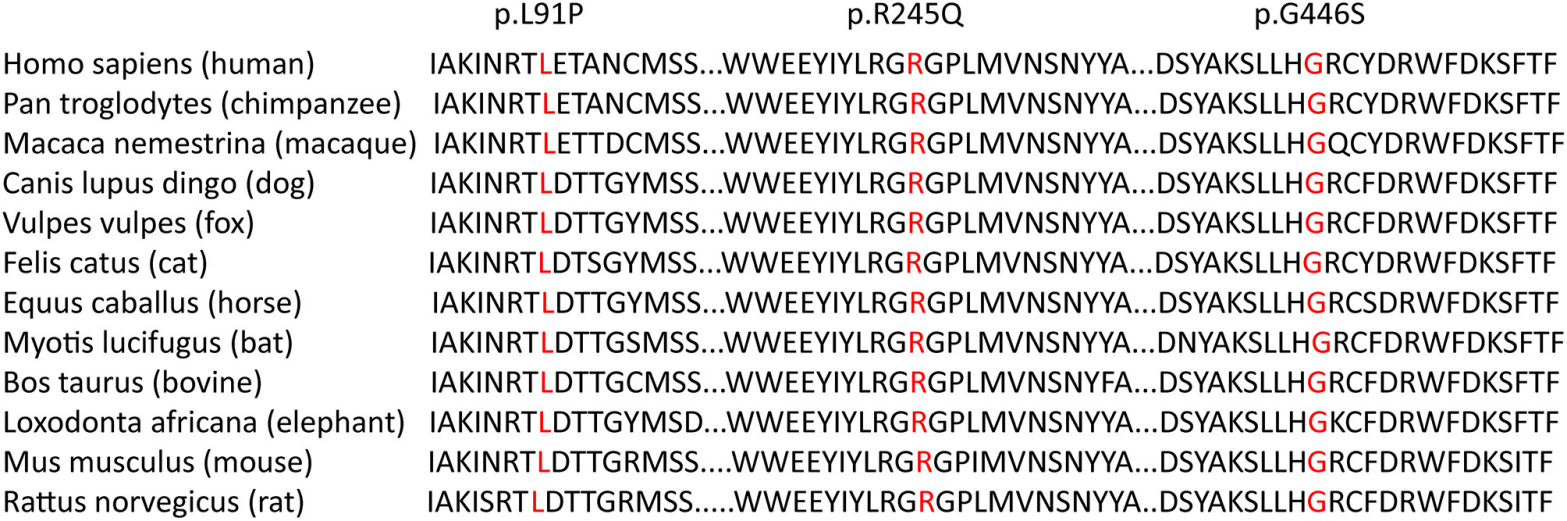
Multiple sequence alignment using ClustalX. Alignment of the CPT1A sequences revealed that the amino acid residues at positions 91, 245, and 446 (highlighted in box) were strictly conserved.

**Figure 3:**
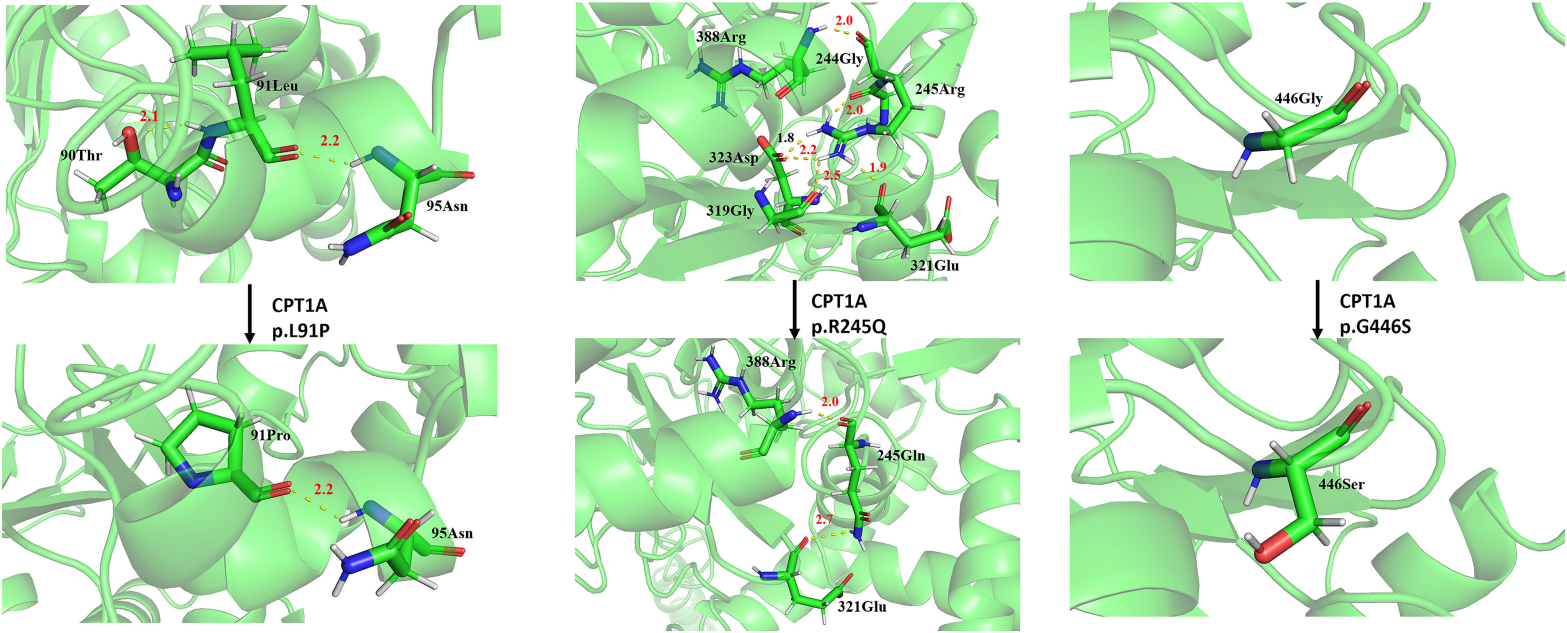
Three-dimensional structural modeling of wild-type and mutant CPT1A proteins. The yellow dashed lines represent hydrogen bonds, and the red numbers represent the hydrogen bond distances. Leu-91 is located in the extracellular segment between two transmembrane domains. The p.L91P variant may affect the quaternary structure of CPT1A by causing the loss of the side chain hydrogen bond with Thr-90, which may result in abnormal folding of CPT1A. p.R245Q is located in the C-terminal catalytic domain, which may affect the quaternary structure of CPT1A by eliminating the side chain hydrogen bonds with Gly-244, Gly-319, and Asp-323, possibly resulting in abnormal folding. p.G446S is located in the C-terminal catalytic domain; the beta turn in the CPT1A secondary structure was changed to a random coil, resulting in an unstable protein.

None of these novel variants were recorded in disease databases such as ClinVar and HGMD. The novel variants were absent or had extremely low allelic frequencies in the dbSNP, ExAC, 1000 Genome, and GnomeAD databases. *In silico* analysis suggested that all three novel variants were potentially pathogenic (Supplementary Table 1). Alignment of the CPT1A sequences revealed that the amino acid residues at positions 91, 245, and 446 were strictly conserved (Figure 2). Protein modeling showed that Leu-91 was located in the extracellular segment between two transmembrane domains; the p.L91P variant may affect the quaternary structure of CPT1A by causing the loss of the side chain hydrogen bond with Thr-90, which may result in abnormal folding. p.R245Q was located in the C-terminal catalytic domain, which may affect the quaternary structure of CPT1A by eliminating the side chain hydrogen bonds with Gly-244, Gly-319, and Asp-323, possibly resulting in abnormal folding. Likewise, p.G446S was located in the C-terminal catalytic domain, and the beta turn in the secondary structure of CPT1A became a random coil, resulting in protein instability (Figure 3). All variants were classified as variants of unknown significance (PM2+PP3) according to the ACMG/AMP guidelines for interpretation of sequence variation.

Although the pathogenicity of these novel variants could not be confirmed, we provided the following evidence supporting an association between these variants and CPT1A deficiency: (i) the variants were inherited from the parents, who were carriers; (ii) the variants were not present or had extremely low allelic frequencies in population databases, altered the strictly conserved amino acid residues of CPT1A, and were predicted to be deleterious by different computational tools; and (iii) protein modeling analysis indicated that the variants could lead to structural damage or CPT1A dysfunction.

## Discussion

Except for in a few specific ethnic groups, CPT1A deficiency seems to be very rare in the general population. This study presented the biochemical, clinical, and genetic characteristics of Chinese patients with CPT1A deficiency, highlighting the value of NBS, and contributing to the body of knowledge needed for early diagnosis and timely intervention of this rare disorder.

The incidence of CPT1A deficiency varies significantly among different ethnic groups. NBS data from Germany, Australia, and the USA showed that the incidence of CPT1A deficiency was as low as 1:750,000 to 1:2,000,000 in 2010 (17). The actual incidence of CPT1A deficiency in China remains unclear. The largest nationwide cross-sectional study using a standardized questionnaire showed that the overall incidence of CPT1A deficiency in mainland China is 1:546,128 (18). We identified two Chinese patients with CPT1A deficiency through our NBS program, which indicated an estimated prevalence of 1:102,388 in the southern Chinese newborn population. This incidence is higher than that reported in previous studies, and this variation may be due to differences in ethnic backgrounds, cut-off value settings, recall criteria, diagnostic methods, and awareness of the disease. It is noteworthy that the availability of NBS is relatively limited, and there may be some patients that cannot be identified by MS/MS-based NBS.

All patients showed elevated C0 levels and/or C0/(C16+C18) ratios, and one patient had a normal C0/(C16+C18) ratio. Therefore, these two screening markers do not necessarily increase at the same time, and any significant increase in one marker should be interpreted as a positive NBS result. Although patients with CPT1A deficiency can be identified by NBS, previous studies have shown that milder forms of CPT1A deficiency can be difficult to diagnose because screening markers may be normalized or only slightly increased during follow-up (19, 20). These patients may be missed or not diagnosed until the patient presents with acute metabolic decompensations. To avoid delayed diagnosis, it has been proposed that all patients with abnormal NBS results suspected of CPT1A deficiency should undergo mandatory *CPT1A* sequencing and/or enzyme analysis (20).

None of the five patients diagnosed *via* NBS had any clinical symptoms, while all of the clinically diagnosed patients presented with a variety of clinical symptoms, indicating that NBS for CPT1A deficiency is beneficial. However, we should note that patients diagnosed using NBS may also develop clinical manifestations when energy demands increase, such as during fasting or febrile illness. Therefore, dietary management and avoidance of prolonged fasting are recommended, and careful follow-up is required to ensure that the patient has a good prognosis.

*CPT1A* is located on chromosome 11q13.3 and contains 19 exons spanning more than 60 kb of genomic DNA. To date, at least 30 *CPT1A* pathogenic variants have been reported. Most pathogenic variants appear to be unique or identified only in a few pedigrees, except c.1436C>T (p.P479L) and c.2129G>A (p.G710E). c.1436C>T (p.P479L) was found to be highly prevalent in northern Canada, Greenland, Siberia, and the Alaska Native population (5, 7, 21). c.2129G>A (p.G710E) was recognized as a founder mutation in the Hutterite population (22, 23). Reports on Chinese patients with CPT1A deficiency are limited, and no hotspot *CPT1A* mutations have been reported to date. The cohort of Chinese patients in our study was characterized by wide allelic heterogeneity, with 18 different variants identified. c.2201T>C (p.F734S) was the most frequent variant, suggesting that c.2201T>C (p.F734S) may be a potential hotspot *CPT1A* mutation in the Chinese population. This variant has not yet been found in other populations; therefore, it could be unique to the Chinese population. The remaining variants were detected only once or twice. Three novel variants were identified, further expanding the mutational spectrum of *CPT1A*. Although computational prediction is helpful for assessing potential pathogenicity, further functional investigations are warranted to confirm the pathogenicity of these novel variants.

In conclusion, the incidence of CPT1A deficiency in Quanzhou, China, was estimated to be 1:102,388. Furthermore, the biochemical, clinical, and genetic characteristics of Chinese patients with CPT1A deficiency documented in this study may facilitate early diagnosis and intervention. Moreover, our findings demonstrated that NBS for CPT1A deficiency is beneficial. This cohort of Chinese patients was characterized by wide allelic heterogeneity. The three novel variants identified in this study expand the mutational spectrum of *CPT1A* in the Chinese population. c.2201T>C (p.F734S), the most frequent variant, may be a potential hotspot *CPT1A* mutation in this population.

## Data Availability Statement

The data presented in the study are deposited in FigShare, accession number 10.6084/m9.figshare.16920841.

## Ethics Statement

The studies involving human participants were reviewed and approved by the Ethical Committee of Quanzhou Maternity and Children's Hospital. Written informed consent to participate in this study was provided by the participants' legal guardian/next of kin. Written informed consent was obtained from the individual(s), and minor(s)' legal guardian/next of kin, for the publication of any potentially identifiable images or data included in this article.

## Author Contributions

WZ performed the data analysis and drafted, and revised the manuscript. YC revised the manuscript. CL, WP, and QF assisted with data collection. YL designed and supervised the research study. All authors contributed to the data analysis and revised and approved the final manuscript for publication.

## Funding

This work was funded by the Natural Science Foundation of Fujian Province (Grant Nos. 2020J01130 and 2021J01538) and the Youth Research Project in the Health System of Fujian Province (Grant No. 2020QNA083).

## Conflict of Interest

The authors declare that the research was conducted in the absence of any commercial or financial relationships that could be construed as a potential conflict of interest.

## Publisher's Note

All claims expressed in this article are solely those of the authors and do not necessarily represent those of their affiliated organizations, or those of the publisher, the editors and the reviewers. Any product that may be evaluated in this article, or claim that may be made by its manufacturer, is not guaranteed or endorsed by the publisher.

## Abbreviations

CPT1A: carnitine palmitoyltransferase 1A
MS/MS: tandem mass spectrometry
NBS: newborn screening
C0: free carnitine
C0/(C16+C18): C0 to free long-chain acylcarnitine
NGS: next-generation sequencing
PCR: polymerase chain reaction
3D: three-dimensional
ACMG/AMP: American College of Medical Genetics and Genomics and the Association for Molecular Pathology.
