# Supplementary material for: Three Novel and One Potential Hotspot CPT1A Variants in Chinese Patients With Carnitine Palmitoyltransferase 1A Deficiency
Source: Front Pediatr. 2021 Nov 12;9:771922. doi: 10.3389/fped.2021.771922 (PMC8633485; doi:10.3389/fped.2021.771922)
Supplement: Supplementary file 1 [file Table_1.docx]

**Table S1.** *In silico* prediction and analysis of novel *CPT1A* variants

| No. | Location | Nucleotide change | Protein change | SIFT^a^ | PolyPhen-2^b^ | PROVEAN^c^ | Mutation Taster^d^ | HGMD^e^ | ClinVar^f^ | LOVD^g^ | dbSNP^h^ | Freq in GnomAD^i^ | Freq in ExAC^j^ | Freq in 1000 Genome^k^ |
| --- | --- | --- | --- | --- | --- | --- | --- | --- | --- | --- | --- | --- | --- | --- |
|  |  |  |  |  |  |  |  |  |  |  |  |  |  |  |
| 1 | Exon 3 | c.272T>C | p.L91P | 0.001 | 0.968 | -4.317 | 0.999 | ND | ND | ND | ND | ND | ND | ND |
| 2 | Exon 7 | c.734G>A | p.R245Q | 0 | 0.943 | -3.302 | 0.999 | ND | ND | ND | rs756976085 | 2.85E-05 | 2.60E-05 | ND |
| 3 | Exon 11 | c.1336G>A | p.G446S | 0.01 | 0.993 | -5.351 | 0.999 | ND | ND | ND | rs749294263 | 2.03E-05 | 1.60E-05 | ND |

ND: no data.

^a^SIFT: <http://sift.jcvi.org/>, ^b^PolyPhen-2: <http://genetics.bwh.harvard.edu/pph2/>, ^c^PROVEAN: <http://provean.jcvi.org/index.php>, ^d^MutationTaster: <http://www.mutationtaster.org/>, ^e^HGMD: <http://www.hgmd.cf.ac.uk/ac/index.php>, ^f^ClinVar: <https://www.ncbi.nlm.nih.gov/clinvar/>, ^g^Leiden Open Variation Database <http://www.lovd.nl/3.0/home>, ^h^dbSNP: <https://www.ncbi.nlm.nih.gov/projects/SNP/>, ^i/j^ExAC/GnomAD: <http://gnomad.broadinstitute.org/>, ^k^1000 Genome Project: http://www.1000genomes.org/.
